# Phytochemical Composition and Anti-Efflux Pump Activity of Hydroalcoholic, Aqueous, and Hexane Extracts of *Artemisia tournefortiana* in Ciprofloxacin-Resistant Strains of *Salmonella enterica* Serotype Enteritidis

**Published:** 2020-01

**Authors:** Marjan KHOSRAVANI, Mohammad Mehdi SOLTAN DALLAL, Mehdi NOROUZI

**Affiliations:** 1 Department of Microbiology, Fars Science and Research Branch, Islamic Azad University, Shiraz, Iran; 2 Department of Microbiology, Shiraz Branch, Islamic Azad University, Shiraz, Iran; 3 Food Microbiology Research Center, Tehran University of Medical Sciences, Tehran, Iran; 4 Divison of Microbiology, Department of Pathobiology, School of Public Health, Tehran University of Medical Sciences, Tehran, Iran; 5 Department of Virology, School of Public Health, Tehran University of Medical Sciences, Tehran, Iran

**Keywords:** *Artemisia tournefortiana*, *Salmonella enteritidis*, Real-time polymerase chain reaction (PCR)

## Abstract

**Background:**

The AcrB efflux pump in Salmonella species plays a significant role in the development of antibiotic resistance in ciprofloxacin-resistant *Salmonella enteritidis*. This study aimed to investigate the anti-efflux pump activity of *Artemisia tournefortiana* extracts among *S. Enteritidis* strains.

**Methods:**

The hydroalcoholic, aqueous, and hexanolic extracts of *A. tournefortiana* were prepared and phytochemical composition of extract was determined using gas chromatography/mass spectrometry (GC/MS) method. After antibiogram, the AcrB efflux pump was detected in ciprofloxacin intermediate and resistant *S. enteritidis* strains using cartwheel and Polymerase chain reaction (PCR) methods. Finally, minimum inhibitory concentrations (MIC) of extracts against *S. enteritidis* strains were evaluated. After treatment of *S. enteritidis* strains with sub-MIC concentrations of extracts, the expression level of AcrB efflux pump gene was evaluated using Real-Time PCR.

**Results:**

Phytochemical analysis of extracts using GC/MS method showed that hexadecanoic acid, ethyl ester (30.7%), and cyclopropane,1-(1-hydroxy-1-heptyl)-2-methylene-3-pentyl (17.8%) were the most dominant volatile components volatile compounds in the extract. The results of antibiogram, cartwheel and PCR methods showed that among 20 strains of *S. enteritidis* that were resistant and intermediate to ciprofloxacin, 16 strains had AcrB efflux pumps. Finally, Real-Time PCR results showed a significant down-regulation of *acrB* gene in *S. enteritidis* strains.

**Conclusion:**

A. *tournefortiana* had anti-efflux activity and this plant can potentially be used as a natural efflux inhibitor for *S. enteritidis* strains.

## Introduction

*Salmonella enterica* Serotype Enteritidis (*S. enteritidis*) are among food-borne pathogenic bacteria that have become resistant to common antibiotics (1). Ciprofloxacin-resistant Salmonella strains are among the most important antibiotic-resistant bacteria that are gradually becoming resistant to all antibiotics (2). Resistance to ciprofloxacin has also occurred following administration of this antibiotic for treatment of infections caused by *S. enteritidis*, reached a resistance level of 100% in some cases (3).

Generally, *S. enteritidis* strains have various resistance mechanisms to antibiotics; one of these is the prevention of intracellular drug accumulation by efflux systems (4). Efflux pumps drain toxic agents like antibiotics out of cells and the presence of these pumps is a faculty of this bacterium to become resistant to antibiotics (5). The Resistance-Nodulation division (RND) efflux system is an important system in *S. enteritidis* and the AcrB efflux pump is a key pump belonging to this family. AcrB can pump out various compounds such as hydrophobic fluoroquinolones (like norfloxacin and ciprofloxacin), bioacids, ethidium bromide (EtBr), and tetracyclines (6). *S. enteritidis* strains that have been subject to inactivation of the AcrB efflux pump have become susceptible to ciprofloxacin (7). Recently, many researchers have attempted to use alternative therapies for treatment of pathogenic bacteria, especially to suppress efflux pumps. For researchers, herbal extracts are an alternative to inactivation of efflux pumps (8). In this research, we studied a medicinal plant native to Iran called *Artemisia tournefortiana*. Artemisia belongs to the Asteraceae (or Compositae) family and includes 200 to 500 species or subspecies as well as five subgenera (9). Artemisia is rich in agents having various effects including anti-inflammatory, anti-tumor, anti-peptic ulcer, diuretic, anti-oxidant, anti-malarial, anti-dyspepsia, anti-proliferative, and gallbladder contraction effects (10,11).

Considering the fact that there has been no study on identification of chemical compounds of the *A. tournefortiana* extract and its anti-efflux activity, the aim of this study was to examine phytochemical composition of the *A. tournefortiana* extract and evaluate its anti-efflux activity against the AcrB pump in ciprofloxacin-resistant *S. enteritidis* strains isolated from clinical samples.

## Methods

### Plant collection and extraction

*A. tournefortiana* was obtained from the Iranian Biological Center with herbarium number 1000632P. Fresh aerial parts of *A. tournefortiana* were air-dried in shade for one to two weeks and the aqueous, ethanolic and haxanic extracts were prepared.

### GC/MS analysis of extract and total phenolic test

GC/MS analysis of *A. tournefortiana* extract was performed using the Agilent 6890 device (USA). The database of the National Institute of Standard and Technology (NIST), which includes over 62,000 templates, was used for the interpretation of GC/MS spectra. Total phenolic content was measured by a spectrophotometer using the Folin-Ciocalteau reagent (11).

### Measurement of quercetin and rutin

In this study, HPLC (Knauer, Germany) analysis with C18 column was used to determine antioxidant compounds of quercetin and rutin. The device was connected to a UV detector, which scanned the 190–400 nm spectrum range. Elution was performed by solution A (glacial acetic acid) and solution B (methanol) as the mobile phase. Gradient slope of solution B and solution A were between 32%–100% and 68%–0% within 35 min, respectively. The detection wavelength was set at 360 nm with a flow rate of 1.3 ml/min. Quercetin and rutin (Sigma) standards were also used.

### Sample collection and identification of Salmonella enteritidis isolates

Overall, 60 *S. enteritidis* strains were isolated from 1200 human stool samples. The samples were collected during a span of six months from hospitals in Tehran (Iran) between Mar-2015 to Sep-2016. Identification of *S. enteritidis* strains was based on microbiological and serological tests. After conducting biochemical tests, serotyping was performed to determine O and H antigens with specific antisera for confirmation of Salmonella strains (Staten Serum Institute, Copenhagen, Denmark).

### Antibiotic susceptibility test

Antibiotic susceptibility test was evaluated using the disc diffusion method according to CLSI (Clinical and Laboratory Standards Institute, 2017) procedure (12). Susceptibility of *S. enteritidis* isolates to Meropenem (10 μg), Imipenem (10 μg), Amoxicillin (10 μg), Ciprofloxacin (5 μg), Ceftazidim (30 μg), Ceftriaxone (30 μg), Cefotaxime (30 μg), Trimethoprim sulfamethoxazole (5 μg), Tetracycline (30 μg), Streptomycin (10 μg), and Chloramphenicol (30 μg) was performed on the Mueller-Hinton agar medium (Merck, Germany). In all experiments,*. S. enteritidis* ATCC 13076.

### Phenotypic detection of AcrB efflux pump using cartwheel method

Cartwheel method was used for phenotypic detection of the efflux pump in *S. enteritidis* isolates. Briefly, ciprofloxacin-resistant and intermediate *S. enteritidis* strains were cultured in 5 ml of appropriate both medium until they reached an optical density (OD) 0.6 at 600 nm wavelength. Subsequently, OD of cultures was adjusted to 0.5 McFarland using PBS. Nutrient agar plates containing different concentrations of EtBr ranging 0–2.5 mg/l were prepared and protected from light. The plates were divided into 8 sections by radial lines (cartwheel pattern) and the selected strains were swabbed on plates starting from the center towards the edges plates. Each plates included one reference strains that serve as positive control. The plates were incubated for 16 h at 37 °C and finally, the fluorescence of each isolate was measured using a suitable source of UV-light such as UV trnasiluminator. The strains were not fluorescent had an efflux pump.

### DNA extraction and AcrB efflux pump detection

DNA extraction was manually performed using the phenol chloroform method. The PCR method was used to detect the *acrB* efflux pump gene in ciprofloxacin-resistant and ciprofloxacin-intermediate isolates of *S. enteritidis*. PCR reaction for the *acrB* gene was performed using forward and reverse primers of 5′ TGAAGACCAGGGCGTATTCCT 3′ and 5′ TTTTTGCGTGCGCTCTTG 3′, respectively, with an initial denaturation temperature of 94 °C for 5 min, denaturation at 94 °C for 30 sec, annealing at 55 °C, extension at 72°C for 30 sec and a final extension for 5 min at 72 °C in 35 cycles (13).

### Determination of minimum inhibitory concentrations (MIC)

Ciprofloxacin-resistant and intermediate strains of *S. enteritidis* were studied for MIC of hydroalcoholic, aqueous, and hexane extracts. MIC test was performed using the microdilution method in microplates based on CLSI standard. This test was done in triplicate using the microdilution method in 96-well plates. Additionally, MIC of extracts was measured in a concentration range of 0.97–250 μg/mL (14).

### MIC of ciprofloxacin

After the detection of ciprofloxacin-resistant and intermediate strains, the strains were subject to MIC test using the dilution method in micro-plates for ciprofloxacin based on CLSI procedure. In order to determine MIC for ciprofloxacin, a range of 0.5–128 μg/mL of concentration was used. Additionally, one well containing bacterial suspension without ciprofloxacin was used as negative control, while one well containing *S. enteritidis* ATCC 13076 and ciprofloxacin and was used as positive control (15).

### MIC of Ethidium bromide (EtBr)

MIC of *EtBr* was performed in triplicate using the microdilution method in 96-well plates. EtBr solution was poured into wells (2–250 μg/ml). An amount of 5μL of ciprofloxacin-resistant and intermediate *S. enteritidis* strains (with 0.5 McFarland concentration) was added to all wells. MIC is considered to be the lowest inhibitory concentration of *EtBr* (16).

### Phenotypic study of active efflux pump

In order to determine efflux pump activity, this test is performed similarly to the MIC method. Briefly, MIC of *EtBr* was determined and 0.5 McFarland of bacterial suspension was added to the wells containing lower than MIC of EtBr. Subsequently, carbonyl cyanide 3-chlorophenylhydrazone hydrazine (CCCP) (with a concentration of 20 μg/mL) was added as an inhibitor of the efflux pump. Activity of the efflux pump is distinguished when MIC of EtBr together with CCCP becomes lower than MIC of EtBr alone (16).

### acrB gene expression analysis

To extract RNA, ciprofloxacin-resistant and intermediate strains were cultured in Nutrient broth for 24 h at 37 °C with sub-MIC concentrations of hydroalcoholic extract. Subsequently, RNA extraction was performed using Trizol (CinnaGen). Then, cDNA synthesis was performed using the Takara kit (Takara, Japan). Finally, concentration of extracted cDNA was determined by NanoDrop. qRT-PCR using a SYBR Green-containing Master Mix (Ampliqon, Denmark) was meant to evaluate the gene expression of the *acrB* efflux pump. Reagents in a final volume of 25 μL included 2 μL of extracted cDNA (100 ng), 10 picomoles of forward and reverse primers,, and 12.5 μL of SYBR Green-containing Master Mix. The temperature program of qPCR was 95 °C for 5 min, 95 °C for 30 sec, and 60 °C for 30 sec in 40 cycles. The 16S rRNA gene was used as internal control. Finally, the relative expression of the *acrB* gene was calculated by the ΔΔ_T_ method. Primers used in this section were *acrB* F 5′-TGAAGACCAGGGCGTATTCCT-3′ and a*crB* R 5′-TTTTTGCGTGCGCTCTTG-3′, as well as 16S rRNA F5’-CGTGTTGTGAAATGTTGGGTTAA-3’and16S rRNA R5’- CCGCTGGCAACAAAGGATAA -3 (13).

### Statistical analysis

Statistical analysis was done using SPSS software (ver. 21, Chicago, IL, USA) and Real-Time PCR data were analyzed using REST software. Values were expressed as means±SD and *P*<0.05 was considered to be statistically significant.

## Results

### Phytochemical analysis of A. tournefortiana extract

GC/MS chromatogram analysis of the extract showed 21 peaks, which revealed the presence of volatile phytochemical compounds in the extract. Twenty-one different constituents were identified by comparing the spectra with NIST library data. Major compounds in the extract included hexadecanoic acid, ethyl ester (30.7%), and cyclopropane, 1- (1-hydroxy-1-heptyl)-2-methylene-3-pentyl (17.8%).

### Total phenolic test

Gallic acid was used as a standard for the evaluation of phenolic compounds. Absorbance of various concentrations of Gallic acid (25, 50, 75, 100, 125, and 150 μg/mL) was determined to draw the calibration curve. The absorbance of samples was read at 765 nm wavelength. Based on the Gallic acid equation (y=0.0067x-0.0194 R^2^=0.9919), total phenol value based on mg Gallic acid/gr was 4.07, 4.41, and 5.06 in the hexane, aqueous, and hydroalcoholic extracts, respectively.

### HPLC results

**Fig. 1:**
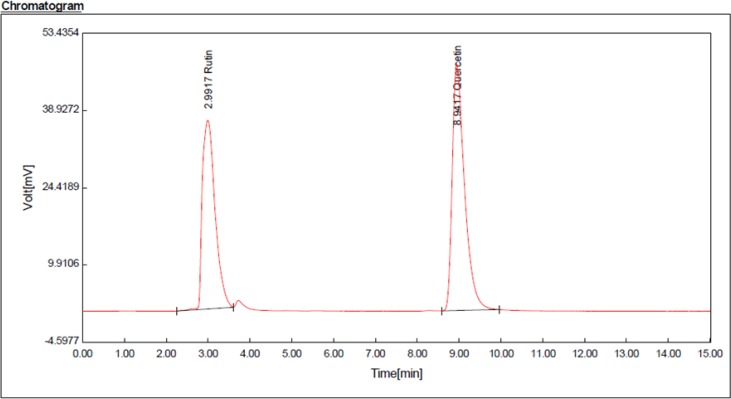
HPLC chromatogram of rutin and quercetin standards

**Fig. 2:**
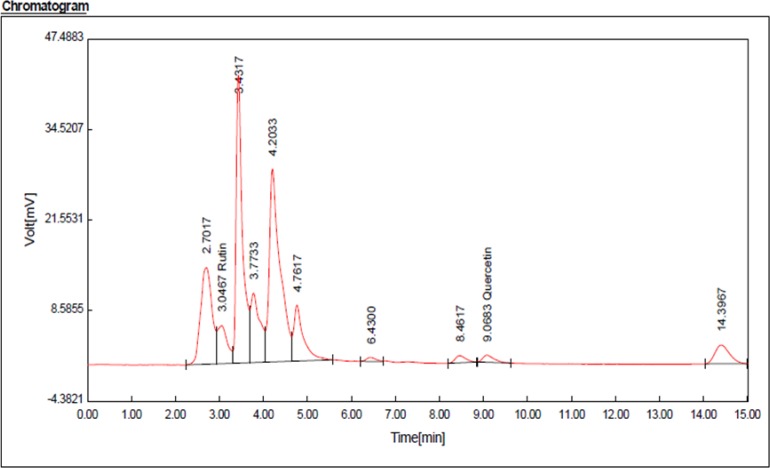
HPLC chromatogram for rutin (Rt: 3.04 min) and quercetin (Rt: 9.06 min) compounds in hydroalcoholic extract of *A. tournefortiana*

According to rutin and quercetin calibration curves (Figs. 1 and 2), rutin value was 3 mg/g and quercetin value was 0.7 mg/g in the hydroalcoholic extract (in triplicate).

### Disc diffusion test results

The highest antibiotic resistance of strains was related to Ciprofloxacin (18%) (Intermediate (15%)) and Sulfamethoxazole trimethoprim (8%); while the lowest antibiotic resistance rate was observed for Ceftriaxone and Cefotaxime (99%), and Chloramphenicol (100%).

### Cartwheel results

**Fig. 3:**
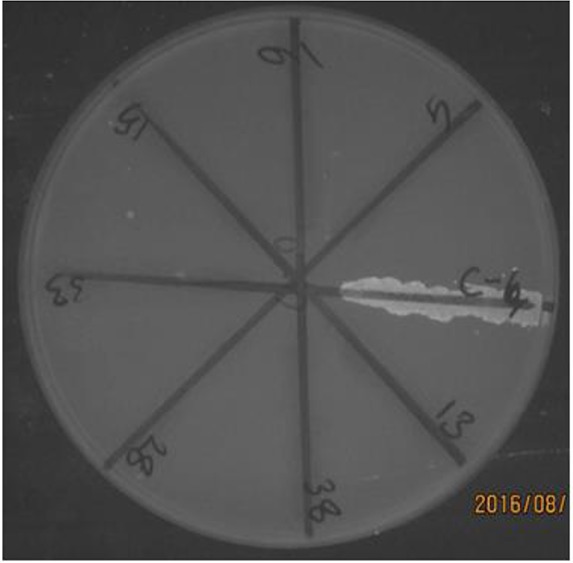
EtBr-agar cartwheel method applied to determination of efflux pump. The strains without efflux pump were observed fluorescent

In this study, ciprofloxacin-resistant strains were examined for detection of efflux pumps using the cartwheel test. All strains resistant and intermediate to ciprofloxacin had efflux pumps (20 samples, 100%) (Fig. 3). The strains with efflux pumps pumped EtBr outwards but those without efflux pumps did not have this ability, which resulted in the entry of EtBr into cells and detection of fluorescence within the cells.

### Detection of acrB gene in S. enteritidis isolates

**Fig. 4:**
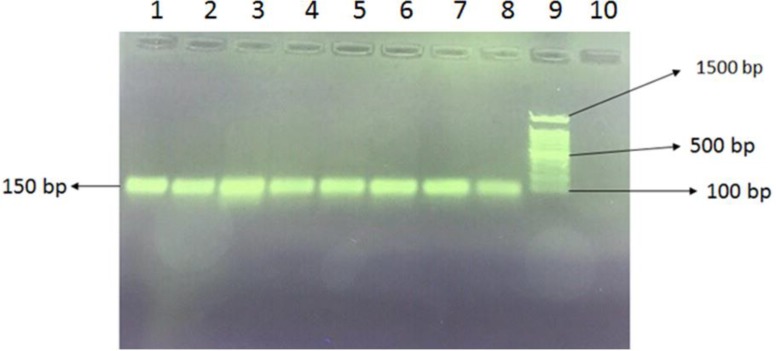
Amplification of *acrB* gene in ciprofloxacin resistant strains. Lane 1–7: acrB gene amplication in salmonella strains, 8: positive control, 9: 100bp plus Ladder, 10: negative control

A specific primer was used for *acrB* efflux pump gene amplification in *S. enteritidis* isolates. The presence of a 150 bp band in PCR products was observed in gel electrophoresis (Fig. 4). The *acrB* gene was detected in 80% of strains (16 samples, ciprofloxacin-resistant and intermediate); there was a significant correlation between the *acrB* gene and resistance to ciprofloxacin among the strains (*P*<0.05).

### MIC of extracts, ciprofloxacin and EtBr

Ciprofloxacin-resistant strains of *S. enteritidis* were subject to 0.97–250 μg/mL concentrations of hydroalcoholic, aqueous, and hexane extracts for 24 h. The hydroalcoholic, aqueous, and hexane extracts had an MIC range of 1.95–3.9, 3.9–31.2 and 3.9–31.2 μg/mL, respectively (Table 1). There was no significant correlation between ciprofloxacin resistance and MIC levels of extracts (*P*>0.05). In this study, MIC of ciprofloxacin, EtBr, and EtBr combined with CCCP, as well as the extract and ethidium bromide was determined for ciprofloxacin-resistant isolates. At MIC of EtBr decreased in the presence of CCCP (Table 2).

**Table 1:** MIC of hydroalcoholic, aqueous, and hexane extracts

***Strain number***	***MIC of hydroalcoholic extract (μg/mL)***	***MIC of aqueous extract (μg/mL)***	***MIC of hexane extract (μg/mL)***
2	3.9	31.2	31.2
3	3.9	15.6	15.6
4	1.95	15.6	15.6
5	1.95	15.6	15.6
6	1.95	31.2	31.2
9	3.9	31.2	31.2
13	1.95	15.6	15.6
14	3.9	15.6	15.6
15	3.9	3.9	3.9
16	3.9	3.9	3.9
19	3.9	3.9	3.9
22	3.9	3.9	3.9
28	3.9	3.9	3.9
31	3.9	7.81	7.81
32	3.9	7.81	7.81
33	1.95	3.9	3.9
38	1.95	3.9	3.9
40	1.95	3.9	3.9
41	1.95	7.81	7.81
66	3.9	7.81	7.81
C + (ATCC13076)	3.9	7.81	7.81

**Table 2:** MIC of ciprofloxacin, ethidium bromide, CCCP, and combination of extract with ethidium bromide in ciprofloxacin-resistant strains

***Strains no.***	***Ciprofloxacin (μg/ml)***	***EtBr (μg/ml)***	***EtBr + CCCP (μg/ml)***	***Extract + EtBr (μg/ml)***
2	0.5	125	62.5	62.5
3	0.5	125	62.5	31/2
4	0.25	125	62.5	62.5
5	1	125	62.5	31/2
6	0. 5	125	62.5	62.5
9	1	125	62.5	62.5
13	0.25	125	62.5	62.5
14	0.5	125	62.5	62.5
15	0.25	125	62.5	62.5
16	1	125	62.5	31/2
19	2	125	62.5	31/2
22	0.5	125	62.5	62.5
28	0.5	125	62.5	62.5
31	2	62.5	31/2	31/2
32	1	62.5	31/2	31/2
33	0.5	125	62.5	62.5
38	1	125	62.5	31/2
40	0.25	62.5	31/2	62.5
41	1	62.5	31/2	31/2
66	2	62.5	31/2	31/2
ATCC13076	2	62.5	31/2	31/2

### acrB gene expression in S. enteritidis strains

**Fig. 5:**
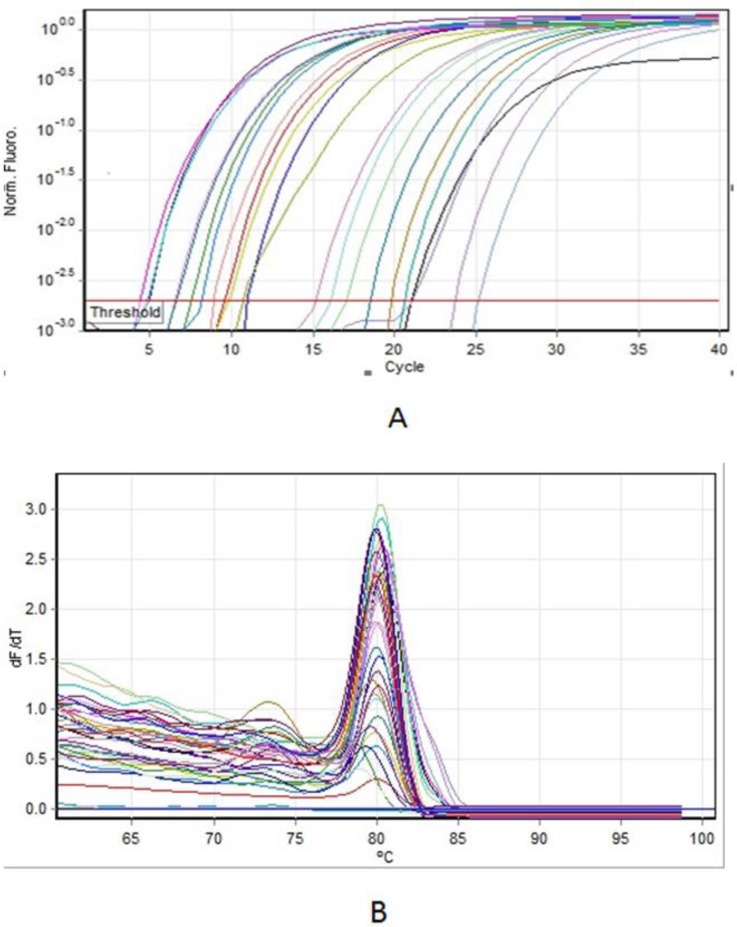
Amplification plot (A) and melting curve (B) of *acrB* gene in *S. enteritidis* ciprofloxacin intermediate and resistant isolates

**Fig. 6:**
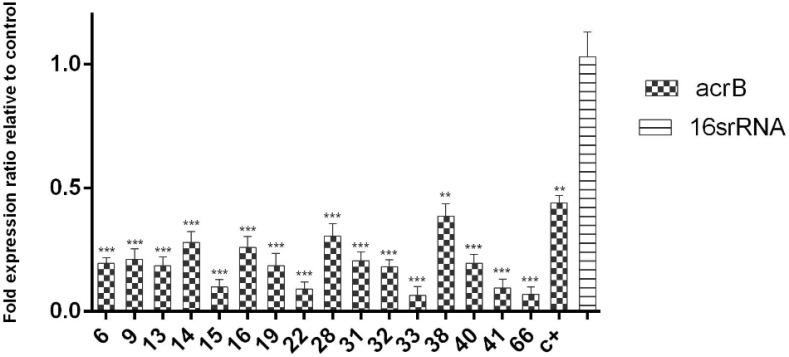
*AcrB* gene expression changes in strains affected by the subMIC concentration of extract. As can be seen, the expression of acrB gene was significantly reduced compared to the control gene of 16S rRNA. *P*<0.05^*^, *P*<0.01^**^, *P*<0.001^***^

Because of higher efficacy of the hydroalcoholic extract as compared to other extracts, the relative gene expression of the *acrB* efflux pump was studied in sub-MIC concentrations of the hydroalcoholic extract in ciprofloxacin-resistant and intermediate isolates using the qRT-PCR method. Specific amplification of the *acrB* gene, non-pairing of primers, and non-amplification of non-specific genes were determined using the melting curve (Fig. 5). Different strains have altered expressions of the *acrB* gene under the influence of hydroalcoholic extract in sub-MIC concentrations. Statistically, there was a significant difference between expression of the *acrB* gene and the 16S rRNA gene (*P*<0.05). In fact, after the treatment of strains with sub-MIC concentration of extracts, gene expression of the *acrB* efflux pump was down-regulated, indicating the inhibitory effect of the efflux pump. The changing expressions of the *acrB* gene in ciprofloxacin-resistant strains are shown in Fig. 6.

## Discussion

After extraction through the maceration method, phytochemical composition of the extract was determined using the GC/MS method. Hexadecanoic acid, ethyl ester (30.7%), and cyclopropane, 1- (1-hydroxy-1-heptyl)-2-methylene-3-pentyl (17.8%) were the main volatile constituents of the extract. Results of total phenolic contents showed that hydroalcoholic extracts have more amount of phenolic contents other than extracts. Results of HPLC showed that each mg of extract has 3 mg rutin and 0.7 quercetin. Additionally, lowest MIC was observed in hydroalcoholic extracts. We can observe the results of antibacterial activity of hydroalcoholic extracts that have direct relations with phenolic compounds. Compounds such as (6,8-Bis-hydroxymethyl-4-isopropyl-7-methylene-bicyclo [3.2.1] oct-1-yl)-methanol, 1,2-benzenedicarboxylic acid, and diisooctyl ester are among the constituents of *A. tournefortiana* extract, which seem to account for the largest portion of anti-microbial and anti-efflux pump effects due to their ring structure and functional groups (17). Various studies have been conducted to determine the phytochemical compounds of different species of the genus Artemisia. The phytochemical compounds of the essential oil studied of *A. tournefortiana* using the GC/MS method, β -thujone (47.0%), sabinene (16.5%), and β-pinene (8.3%) were the most important constituents of *A. tournefortiana* essential oil from Iran (18). Differences in types of compounds detected in *Artemisia* species seem to be due to geographic area, place of growth, and types of plant cultivation.

Results of HPLC show that flavonoids like rutin and quercetin were sufficient in the extract. More studies were reported flovonoieds such as quercetin, rutin, hesperidin and catechin have efflux inhibitory activity and flavonoids are one of the reasons for inhibition of the efflux pump in *S. enteritidis*. One possible mechanism for relationship of flavonoids and efflux inhibition can be suppression of the AcrB efflux pump.

In future studies, by isolating and purifying flavonoids of the plant, they can be investigated for inhibiting the efflux pump. In case of positive results, they can be used in combination with antibiotics to treat the disease caused by ciprofloxacin-resistant *S. enteritidis*.

Another objective of this study was to investigate anti-efflux pump effects of the *A. tournefortiana* extract in ciprofloxacin-resistant clinical isolates of Salmonella. Efflux pumps of *S. enteritidis* strains are among the most important reasons for resistance to antibiotics. In *S. enteritidis*, the AcrB efflux pump has a significant role in resistance to ciprofloxacin, which is a therapeutic target for *S. enteritidis* strains. Over-expression, degradation, and elimination of the efflux pump may affect resistance to various antibiotics in this bacterium. Purification of efflux pump proteins and laboratory study of inhibitory properties of different compounds of the extract can accurately reflect anti-efflux pump effects. Nowadays, researchers are attempting to find natural compounds to inhibit efflux pumps in bacteria; hence, plant extracts are one of the natural choices for inhibiting efflux pumps (19). In this study, *S. enteritidis* strains with AcrB pumps were identified by phenotypic and genotypic methods (cartwheel and PCR, respectively). In the cartwheel method, strains with AcrB efflux pumps pumped out the substrate (EtBr), but those without AcrB pumps were not able to pump the EtBr and were observed as fluorescent bacteria. Moreover, strains with AcrB efflux pumps were confirmed using PCR as a molecular method. There are many reports on efflux inhibition in Salmonella strains by plant extracts. The efflux inhibitory activity of *Phyllanthus emblica* extract was investigated on Salmonella typhimurium strains. The extract has an inhibitory effect on efflux pumps (20).

In the next step, after treatment of ciprofloxacin-resistant isolates harboring the *acrB* gene with sub-MIC concentrations of the extract, gene expression of the AcrB efflux pump was analyzed by Real-Time PCR to evaluate anti-efflux pump effects of the extract. After treatment of strains with a sub-MIC concentration of the extract, expression of the *acrB* gene was significantly reduced as compared to the reference 16S rRNA gene, which indicates significant anti-efflux pump effects of the extract. Inhibitory effects of the extract for efflux pumps varied in different strains, so that some strains showed a higher reduction in the expression of the *acrB* gene; in fact, the extract had a greater anti-efflux pump impact. Compounds with a ring structure and functional groups in the extract of this plant have anti-efflux pump activity. In vitro studies have shown that natural efflux inhibitors can restore the efficacy of an antibiotic by rendering a resistant bacteria susceptible to the antibiotic and decrease the chance of resistance development when used in combination with the antibiotics (21).

By comparing the results of our study with other research studies, natural compounds, particularly herbal materials, have the potential to inhibit efflux pumps. The Artemisia extract be used, together with other therapeutic antibiotics, to treat drug-resistant infections caused by *S. enteritidis*.

## Conclusion

Considering the anti-efflux pump effects of the extract of *A. tournefortiana* plant species, further studies be conducted on biological properties of compounds contained in this plant in order to determine the medical significance of this plant as an inhibitor of efflux pumps and ultimately, as a promising pharmacological supplement for pharmaceutical companies.

## Ethical considerations

Ethical issues (Including plagiarism, informed consent, misconduct, data fabrication and/or falsification, double publication and/or submission, redundancy, etc.) have been completely observed by the authors.
